# Prospective randomized study on the efficacy of three-dimensional reconstructions of bronchovascular structures on preoperative chest CT scan in patients who are candidates for pulmonary segmentectomy surgery: the PATCHES (Prospective rAndomized sTudy efficaCy of tHree-dimensional rEconstructions Segmentecomy) study protocol

**DOI:** 10.1186/s13063-023-07600-w

**Published:** 2023-09-16

**Authors:** Francesco Zaraca, Andreas Kirschbaum, Marco Damiano Pipitone, Luca Bertolaccini, The PATCHES study group, Firas Abu Akar, Giorgio Cannone, Mahmoud Ismail, Marcelo Jiménez, Marko Kostic, Calvin S.H. Ng, Reinhold Perkmann, Elena Priscindaro, Lorenzo Spaggiari, Paula Ugalde

**Affiliations:** 1 Department of Vascular and Thoracic Surgery, Regional Hospital, Bolzano, Italy; 2 Department of Visceral, Thoracic and Vascular Surgery, University of Marburg, Marburg, Germany; 3 Department of Thoracic Surgery, IEO, European Institute of Oncology IRCCS, Via Ripamonti 435, 20141 Milan, Italy

**Keywords:** 3D reconstruction, Segmentectomy, Lung cancer

## Abstract

**Introduction:**

Pulmonary segmentectomy, when combined with hilar and mediastinal lymphadenectomy, is currently considered the gold standard treatment for early-stage lung tumors (NSCLC) smaller than 2 cm in diameter. The preoperative planning for segmentectomies usually includes a contrast-enhanced CT with 2D reconstructions (axial, coronary, and sagittal). Recent technological advances allow 3D (volume rendering) reconstructions of preoperative CT scans, intended to improve the surgeon’s understanding of the segmental anatomy.

The study aims to investigate the added value of 3D reconstruction in enhancing the surgeon’s understanding of anatomical structures, thus facilitating surgical planning and improving oncological outcomes.

**Methods and analysis:**

This is a prospective, randomized, controlled study.

Patients will be randomized into two groups:

1. Group 2D: the preoperative workup for these patients will consist of a contrast-enhanced chest CT with two-dimensional (2D) reconstructions (axial, coronary, and sagittal);

2. Group 3D: the preoperative workup for these patients will consist of a contrast-enhanced chest CT with two-dimensional (2D) reconstructions (axial, coronary, and sagittal) and a 3D reconstruction (volume rendering) of the same chest CT employing dedicated software.

The primary endpoints will be negative margin (R0) resection rate, resection margin (staple line-to-tumor distance), and thoracotomy conversions.

We will use Fisher’s exact test for binary outcomes and Mann–Whitney *U* test for continuous outcomes. For subgroup analyses, we will use regression. Multivariable analyses will be based on logistic regression for binary outcomes and linear regression for continuous outcomes.

**Ethics and dissemination:**

The protocol and the model informed consent forms have been reviewed and approved by the ethics committee (N.: 1–2023) concerning scientific content and compliance with applicable research and human subject regulations.

A Subcommittee on Publications was established to review all publications and report its recommendations to the steering committee. The anonymized participant-level dataset and statistical code for generating the results will not be publicly available.

**Trial registration:**

The protocol was registered at ClinicalTrials.gov (ID: NCT05716815; Prospective rAndomized sTudy efficaCy tHree-dimensional rEconstructions Segmentectomy - Full-Text View - ClinicalTrials.gov). Jan 19, 2023.

**Supplementary Information:**

The online version contains supplementary material available at 10.1186/s13063-023-07600-w.

## Introduction

### Background and rationale

#### Introduction

Lung cancer remains the leading cause of cancer mortality worldwide [1, 2]. Although lung cancer treatment today includes effective, innovative treatments, surgical resection is still the gold standard in the early stages [3, 4]. Minimally invasive thoracic surgery (MITS), such as video-assisted thoracoscopic surgery (VATS) and robotic thoracoscopic surgery (RATS), has achieved remarkable results in the treatment of early-stage non-small cell lung (NSCLC) [5–7] and is now preferred to traditional thoracotomic surgery. Segmentectomy for early-stage lung cancer has been shown to have an excellent long-term prognosis because it removes the neoplastic lesion and protects lung function [8, 9], and when combined with hilar and mediastinal lymphadenectomy, it can achieve a satisfactory oncological outcome [10, 11]. Recently, JCOG0802 confirmed that segmentectomy is the standard procedure for stage IA lung cancer (tumor diameter ≤ 2 cm; consolidation-to-tumor ratio > 0.5) [12].

Segmentectomy, defined in accordance with Oziumi H. et al. [13], requires the surgeon to know the location of the tumors to be removed, i.e., the anatomy of lobes, segments, bronchi, arteries, and veins to be resected. Sublobar resection is more technically demanding than a lobectomy because the anatomy of the pulmonary segments is more complex. Furthermore, vascular and bronchial variations of segments are common [14].

#### Mechanisms

Detailed preoperative planning and simulations of anatomical sub-lobar resection using 3D technology could contribute significantly to a safer operation. With the development of imaging technology, such as multidetector computed tomography (MDCT) and three-dimensional computed tomography bronchography and angiography (3D-CTBA), two-dimensional (2D) images can be converted into three-dimensional (3D) images [15, 16].

#### Existing knowledge

Recently, some retrospective studies [17–22] demonstrated that before performing minimally invasive (MI) lung segmentectomy, 3D reconstruction might guarantee margin- and disease-free resections, reduces thoracotomy conversions and intraoperative blood loss, shortens the operation time, and prevents postoperative air leakage, reducing hospitalization times and costs.

#### Need for a trial

3D reconstruction has long been widely used in vascular surgery departments to plan any endovascular repair procedure of the abdominal or thoracic aorta. A randomized trial is needed to demonstrate the utility and efficacy of using 3D reconstruction even before MI lung segmentectomy.

#### Rationale

Today, conventional computed tomography with 2D reconstruction is the gold standard method for planning MITS. With advances in imaging technology, a 3D reconstruction could improve surgeon practice, patient outcomes, and safety [23, 24].

#### Choice of comparator

The comparison group in our study is mandatory because it represents the current standard that all surgeons perform—patients with standard 2D CT reconstruction.

## Objectives

### Research hypothesis

3D reconstruction is superior to 2D for MI lung segmentectomy planning.

### Primary objective

The primary objective is to determine if 2D + 3D reconstruction compared to 2D improves the oncological radicality of sublobar resections and prevents major intraoperative complications.

### Secondary objectives

The secondary objective is to assess the efficacy of 2D + 3D reconstruction in reducing minor intraoperative complications.

### Trial design

The PATCHES trial is designed as a randomized, controlled, multicenter superiority trial with two parallel groups. Randomization will be performed as block randomization with a 1:1 allocation.

## Methods: participants, interventions, and outcomes

**Fig. 1:**
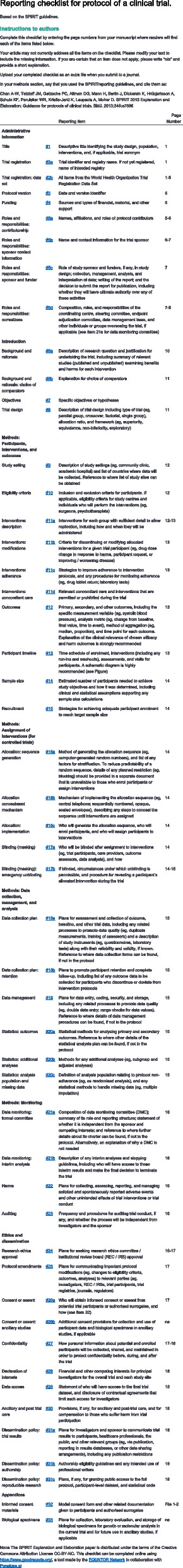
SPIRIT reporting checklist

**Fig. 2:**
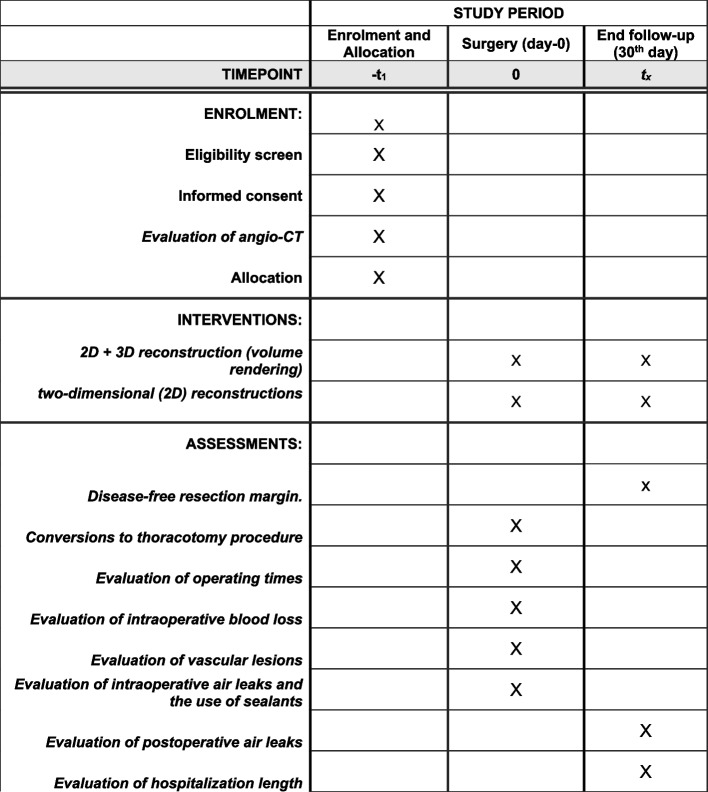
SPIRIT figure for the schedule of enrolment, interventions, and assessments

For the drafting of the article, we used the SPIRIT reporting guidelines (Figs. 1 and 2) [25].

### Study setting

#### Country selection

Ten international high-volume thoracic surgery departments currently participate in the study design. We have included three continents: Europe (Italy, Germany, Belgium), Asia (Hong Kong and Palestine), and the USA (Boston, MA). We selected centers with significant cultural differences to demonstrate the reproducibility of the proposed intervention.

#### Definition of community

To achieve the required sample size with the desired power, the number of segmentectomies at the sites must be high enough. We chose the participating sites so that the average number of annual MI procedures across all communities in the study could reach at least 20 patients per site. Our final selection of sites combines university centers (Marburg, Germany; Salamanca, Spain; Belgrade, Serbia, Harvard Boston, USA; Leuven, Belgium; Hong Kong, China) and hospital centers (Bolzano, Italy, IEO Milan, Italy, Jerusalem, Palestine, Potsdam, Germany).

We e-mailed the trial protocol to recruit participating sites to an extensive list of MI thoracic surgery centers worldwide. The centers listed above have decided to join and have received the relevant material and the approval of the ethics committee of the coordinating center. After approval by each ethics committee, the site will begin enrolling patients.

### Eligibility criteria

Patients (or a representative) must provide written, informed consent before any study procedures occur.

Inclusion criteria

Patients eligible for the trial must comply with all the following at randomization:

- Segmentectomy performed through a MI approach (VATS or RATS)
- Pathologically proven NSCLC on the resected specimen
- Age ≥ 18

Exclusion criteria

- Prior homolateral cardiothoracic surgery
- Allergy or any other contraindication to iodinated contrast media
- Segmentectomy performed through an open approach (thoracotomy)
- Histology different than NSCLC
- Pregnancy

### Interventions

Eligible patients will be randomized in equal proportions in 2 groups. In 50% of patients, the preoperative study of anatomical structures will be performed with the standard method of chest CT evaluation using classic two-dimensional reconstructions (axial, coronary, and sagittal). In the remaining 50% of patients, in addition to the 2D reconstruction, a 3D reconstruction (volume rendering) of the vessels and bronchi of the chest CT will be performed using special software to have a more profound knowledge of the anatomical structures.

3D reconstructions can be performed through the use of Horos (Osirix), a free, open-source medical imaging software program. If necessary, trial participants can change the software. The 3D reconstruction is semi-automatic. After a short learning curve, if preferred, it will take around 45 min.

### Outcomes (see supplementary file for outcome definition)

Primary outcomes measures

- Disease-free resection margin. This measure of success was selected because it was considered clinically and oncologically relevant after segmentectomy and represented a prerequisite and necessary quality control. A disease-free resection margin > 2 cm is mandatory, and a significant result could improve clinical practice and therapeutic outcomes.
- Evaluation of conversions from minimally invasive to thoracotomy procedure. It is still unclear whether preoperative 3D anatomical reconstruction can reduce the incidence of major intraoperative events, which usually require a traditional thoracotomy. This measure of success, if significant, will increase patient safety.

Secondary outcomes measures

- Evaluation of operating times
- Evaluation of intraoperative blood loss
- Evaluation of vascular lesions
- Evaluation of intraoperative air leaks and the use of sealants
- Evaluation of postoperative air leaks
- Evaluation of postoperative hospitalization length

### Participant timeline

Patients with tumors < 2 cm in diameter and suspicion of malignancy will undergo CT and be discussed at the Multidisciplinary Board of Thoracic Oncology. Patients selected for MI segmentectomy will conduct an informational interview and possibly sign the consent to the study. Patients who agreed to participate will be randomized into two groups:

- Group 1: 2D + 3D reconstruction
- Group 2: 2D reconstruction (control group)

**Fig. 3:**
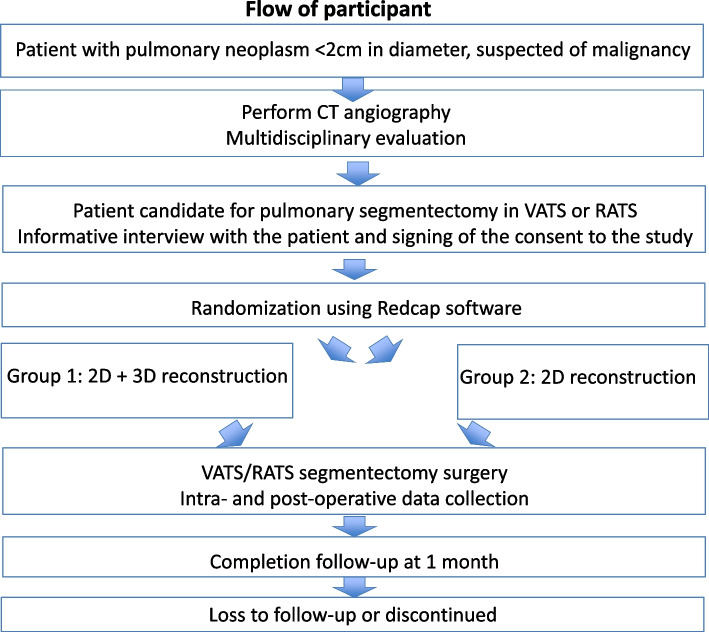
Flow of participants

Subsequently, a database will prospectively collect intraoperative and postoperative data. The follow-up will be closed one month after surgery (Fig. 3).

### Sample size

No data in the current literature was found about the other primary outcome margin- and disease-free resection. Therefore, the sample size was calculated based on the primary outcome conversions from minimally invasive to thoracotomy procedure.

To calculate sample size, we use data about conversion derived from previous literature [17–21]. The conversion rate for patients in the 2D group is estimated to be 9.4% [19]; the retrospective study has shown a reduction to 2.6%. Therefore, the required sample size to confirm superiority was 274, with a power of 80%, a one-sided significance level of 0.05, an accrual period of 3 years, and a follow-up period of 1 month. The planned total sample size was set at 288 patients to account for loss at follow-up.

Systematic methods such as reminders to contact patients, make appointments, and monitor retention will be employed to improve participant retention and will not expenses for follow-up visits and procedures.

We have used for the sample size determination the R software (package pwr) [26].

Two pre-specified interim analyses were conducted after the accrual of half of the planned patients and after accrual completion. To keep the study at a 5% one-sided significance level, we used the Lan–DeMets alpha-spending function [27] with an O’Brien–Fleming approach [28].

### Recruitment

The strategy to achieve adequate participant enrolment and the target sample size was to involve ten participating centers and extend the enrolment period to 3 years.

## Methods: assignment of interventions

### Allocation

All patients who consent to participate and meet the inclusion criteria will be randomized. Participants will be randomly assigned to either a control or experimental group with a 1:1 allocation in accordance with a computer-generated randomization schedule, stratified by participating center, using blocks of a fixed large size. The block size will not be disclosed to ensure concealment.

Randomization management is managed centrally by the REDCap Randomization Module software and is based on an allocation table created by the data analyst (MDP). The allocation table and the details about the restricted randomization (e.g., blocking size) are concealed in a separate document that is unavailable to trial implementers until the end of the study.

Each time a new patient is enrolled and entered in the database by the study coordinator of a center, REDCap will check the allocation table and assign that subject’s randomization field value, which will be derived from the next match in the table based upon the criteria (participating center).

### Blinding (masking)

The trial participants will remain blinded to the group to which they belong to avoid any decision to withdraw from the trial. Even the pathologist does not know the assignment. The measures of outcomes are objective measures; this avoids ascertainment bias. However, outcome assessors and data analysts are kept blinded to the assignment. It is not possible to mask the assignment to the surgeons. This could induce a performance bias; it will be discussed in the limitations of the study.

### Data collection

The measures of outcomes will be collected by medical or nursing staff based on official patient records. Alerts will be sent via e-mail, marking the phases of entering the outcomes on the REDCap software concerning the intervention date. Any data upload delay of more than 2 months will be disclosed in the study. Primary outcomes are not allowed to be missing. The presence of the missing data will be communicated to both the coordinator of the participating center and the data managers. Reasons for missing data will be recorded.

Study data will be collected and managed using REDCap electronic data capture tools hosted at Regional Hospital, Bolzano, Italy [29, 30]. REDCap (Research Electronic Data Capture) is a secure, web-based software platform designed to support data capture for research studies, providing (1) an intuitive interface for validated data capture, (2) audit trails for tracking data manipulation and export procedures, (3) automated export procedures for seamless data downloads to standard statistical packages, and (4) procedures for data integration and interoperability with external sources.

Access to the study data will be restricted, and participating centers will only have access to their data. Incremental data back-ups will be performed daily.

Data collection takes place at regular intervals after surgery (1, 15, 30 days). Regular reminders will be sent to the coordinator of the participating center for operative and postoperative data to be entered in due course.

Participating centers are encouraged to enroll and randomize eligible patients no sooner than 30 days before the scheduled surgery date to minimize the number of ineligible patients after randomization or deviate from the intervention. Participants are retained in the group to which they were originally allocated (“as randomized” analysis). However, patients with missing data on primary outcomes will be excluded from the study. The patient exclusion will be recorded and made explicit in the results.

### Statistical methods

We do not expect a relevant degree of missing data. Methods of multiple imputations (bagged trees imputation) will be used to calculate missing values.

Multivariable analyses will be based on logistic regression for binary outcomes and linear regression for continuous outcomes.

We will examine the residual to assess model assumptions and goodness-of-fit. *P*-values will be reported to three decimal places, with *p*-values less than 0.001 reported as *p* < 0.001. All analyses were performed using the R Statistical Software (4.2.2; R Core Team 2022). We will use 2-sided *p*-values with alpha ≤ 0.05 significance level for all tests.

## Methods: monitoring

### Formal committee

A data monitoring committee (DMC) has been established. The DMC is independent of the study organizers. During the study’s recruitment period, interim analyses are not scheduled.

The role of the DMC is to advise the TSC (trial steering committee) if will be detected relevant adverse events in the experimental or standard group.

The chair of DMC is LB, with MDP, GC, and AK.

### Harms

An adverse event is not really expected in our study for the type of study we conduct. The primary outcomes are disease-free surgical margins and thoracotomy conversions, which we will collect to verify any differences between the two groups. Adverse events, if any, will be collected after the subject has provided consent and enrolled in the study.

Unexpected harms will be collected systematically through a specific request to each participant at each follow-up visit. Harms will be collected and analyzed using standard grading criteria according to the Common Terminology Criteria for Adverse Events (CTCAE) [31].

Any adverse event that occurs between study enrollment and hospital discharge will be reported to the local IRB (institutional review board).

### Auditing

The trial management committee (TMC) will perform weekly visual cross-validation of the data for complex errors and regular on-site monitoring; they will also monitor data quality and completeness.

At the beginning of the trial, the TMC will conduct a tutorial on the web-based data entry system and how to obtain an effective three-dimensional reconstruction of the preoperative CT of vessels and bronchi.

They will review the feedback forms with all participating centers monthly.

The process will be completely independent of the investigators and the sponsor.

## Ethics and dissemination

This protocol and the model informed consent forms contained in Additional files 1, 2, and 3 have been reviewed and approved by each ethics committee (EC) concerning scientific content and compliance with applicable research and human subject regulations on January 18, 2023.

The ethical review bodies will also review and approve the protocol, site-specific informed consent forms, participant education, recruitment materials, other requested documents, and any subsequent modifications.

After initial review and approval, the responsible local EC will review the protocol only if the TMC makes modifications to the protocol. At the end of the trial, the PI will inform the local CE and present a detailed report of the results.

Each participating center submitted a request for approval by its ethics committee.

### Protocol amendments

Any protocol changes that may impact study conduct will be discussed with the entire PATCH study group and possibly submitted and approved by the local CE before implementation and notified to other participating centers.

### Consent or assent

The investigator will explain to each patient (or legal representative) the nature of the study, its purpose, procedures, expected duration, potential risks and benefits, and any inconvenience it may cause. Each patient will be informed that participation in the study is voluntary, that he can withdraw at any time, and that withdrawal of consent will not affect his medical treatment or subsequent relationship with the treating physician. Informed consent will be given by written communication, using non-technical language. The patient is given time to read and understand the consent before signing it; a copy of the document will be delivered. If the subject cannot read, the presentation can be made orally, and if he cannot sign the document, the legal representative will provide the signature, mentioning that the patient could not read or sign the documents. No patient can enter the study without having been informed and given consent.

Patients will also receive information sheets.

No additional studies will be done with the data collected in this trial.

### Confidentiality

Data for this study will be collected in a REDCap® (Research Electronic Data Capture) database.

In compliance with the ICH/GCP (International Conference on Harmonisation/Good Clinical Practice) guidelines, the investigator/institution will maintain all eCRFs and all source documents that support the data collected from each subject, as well as all study documents as specified in ICH/GCP Sect. 8, Essential Documents for the Conduct of a Clinical Trial, and all study documents as specified by the applicable regulatory requirement(s).

The promoter/study coordinator will take measures to prevent these documents’ accidental or premature destruction.

If the investigator retires, relocates, or, for other reasons, withdraws from the responsibility of keeping the study records, custody must be transferred to a person who will accept the responsibility. Under no circumstance shall the promoter/study coordinator relocate or dispose of any study documents before obtaining written approval from the center providing the data. Each center has free access to its data through the database and is free to extract them from the database at any time.

### Declaration of interests

The investigators have no conflict of interest to declare.

### Access to data

A dedicated committee will be set up to prevent data sharing from the entire Database of Access to Data. The scientific director of the study and the participating centers’ coordinators are part of the AC.

Each center has free access to its data through the database and is free to extract them from the database at any time. The TMC has access to the entire database, and no contractual agreements limit that access for investigators.

### Ancillary and post-trial care

This study should provide evidence of the efficacy of a preoperative three-dimensional reconstruction of chest CT vessels and bronchi and should be added to clinical practice.

No insurance has been arranged to cover non-negligent damages associated with the protocol because it is unnecessary.

### Dissemination policy

A subcommittee on publications will be established to review all publications and report its recommendations to the steering committee.

The investigators will communicate trial results via open-source publications, and there are no restrictions.

The anonymized participant-level dataset and statistical code for generating the results will not be publicly available.

### Authorship

PI and DMs will be the lead authors of the material derived from this study. No professional writers are expected.

### Biological specimens

No biological materials will be collected throughout the trial.

### Supplementary Information

**Additional file 1. **Informed consent.

**Additional file 2. **Information sheet.

**Additional file 3. **Supplementary file.
