# Correction: Prospective randomized study on the efficacy of three-dimensional reconstructions of bronchovascular structures on preoperative chest CT scan in patients who are candidates for pulmonary segmentectomy surgery: the PATCHES (Prospective rAndomized sTudy efficaCy of tHree-dimensional rEconstructions Segmentecomy) study protocol

**DOI:** 10.1186/s13063-023-07671-9

**Published:** 2023-10-03

**Authors:** Francesco Zaraca, Andreas Kirschbaum, Marco Damiano Pipitone, Luca Bertolaccini, The PATCHES study group

**Affiliations:** 1 Department of Vascular and Thoracic Surgery, Regional Hospital, Bolzano, Italy; 2 Department of Visceral, Thoracic and Vascular Surgery, University of Marburg, Marburg, Germany; 3 Department of Thoracic Surgery, IEO, European Institute of Oncology IRCCS, Milan, Italy

**Correction: BMC Trials 24, 594 (2023)**

**https://doi.org/ 10.1186/s13063-023-07600-w**

Following publication of the original article [1], we have been notified of a few mistakes presented in the affiliations for the PATCHES study group, in the Acknowledgements section.

- Originally published affiliations:

Firas Abu Akar, MD [1] Giorgio Cannone, MD [2] Mahmoud Ismail, MD [3] Marcelo Jiménez, MD [4] Marko Kostic, MD [5] Calvin S.H. Ng, MD [6] Reinhold Perkmann, MD Elena Priscindaro, MD [7] Lorenzo Spaggiari, MD Paula Ugalde, MD [8]

1 Department of Cardiothoracic Surgery, Al-Makassed Charitable Society Hospital, East Jerusalem, Palestine.

2 Department of Vascular and Thoracic Surgery, Regional Hospital, Bolzano, Italy.

3 Klinikum Ernst von Bergmann, Potsdam, Germany.

4 Department of Thoracic Surgery, Salamanca University Hospital, Salamanca, Spain.

5 Clinic for Thoracic Surgery, University Clinical Center of Serbia, University of Belgrade, Belgrade, Serbia.

6 The Chinese University of Hong Kong, Prince of Wales Hospital, Shatin, New Territories, Hong Kong, China.

7 Department of Thoracic Surgery, UZ Leuven, Belgium.

8 Division of Thoracic and Cardiac Surgery Brigham and Women’s Hospital, Harvard University, USA.

- Corrected affiliations:

Firas Abu Akar, MD [4], Giorgio Cannone, MD [1], Mahmoud Ismail, MD [5], Marcelo Jiménez, MD [6], Marko Kostic, MD [7], Calvin S.H. Ng, MD [8], Elena Priscindaro, MD [9], Lorenzo Spaggiari, MD [3, 10], Reinhold Perkmann, MD [1], Paula Ugalde, MD [11]

1 Department of Vascular and Thoracic Surgery, Regional Hospital, Bolzano, Italy.

2 Department of Visceral, Thoracic and Vascular Surgery, University of Marburg, Marburg, Germany.

3 Department of Thoracic Surgery, IEO, European Institute of Oncology IRCCS, Milan, Italy.

4 Department of Cardiothoracic Surgery, Al-Makassed Charitable Society Hospital, East Jerusalem, Palestine.

5 Klinikum Ernst von Bergmann, Potsdam, Germany.

6 Department of Thoracic Surgery, Salamanca University Hospital, Salamanca, Spain.

7 Clinic for Thoracic Surgery, University Clinical Center of Serbia, University of Belgrade, Belgrade, Serbia.

8 The Chinese University of Hong Kong, Prince of Wales Hospital, Shatin, New Territories, Hong Kong, China.

9 Department of Thoracic Surgery, UZ Leuven, Belgium.

10 Department of Oncology and Hemato-Oncology, University of Milan, Milan, Italy.

11 Division of Thoracic and Cardiac Surgery Brigham and Women’s Hospital. Harvard University. USA.

The original article has been corrected.
