# Supplementary material for: Prospective randomized study on the efficacy of three-dimensional reconstructions of bronchovascular structures on preoperative chest CT scan in patients who are candidates for pulmonary segmentectomy surgery: the PATCHES (Prospective rAndomized sTudy efficaCy of tHree-dimensional rEconstructions Segmentecomy) study protocol
Source: Trials. 2023 Sep 16;24:594. doi: 10.1186/s13063-023-07600-w (PMC10504737; doi:10.1186/s13063-023-07600-w)
Supplement: Supplementary file 1 — Additional file 1. Informed consent. [file 13063_2023_7600_MOESM1_ESM.docx]

| **Abteilung Gefäß- und Thoraxchirurgie**  **Vascular and Thoracic Surgery Division**  **Head physician**  **Dr. R Perkmann** |  |
| --- | --- |

**INFORMED CONSENT**

PROSPECTIVE RANDOMIZED STUDY ON THE EFFICACY OF THREE-DIMENSIONAL RECONSTRUCTIONS OF BRONCHOVASCULAR STRUCTURES ON PREOPERATIVE CHEST CT IN PATIENTS WHO ARE CANDIDATES FOR PULMONARY SEGMENTECTOMY SURGERY

I, the undersigned ______________________________ born on the __/___/_____(dd/mm/yyyy) in_______________, residing at ________________________City____________State _______

Post Code ______ tel./email______________________________________

With my signature I declare that I

- have read the information leaflet and spoken to my doctor
- voluntarily submit to the study; At any time, I can withdraw from the study without affecting the treatment of my pathology

I also agree:

- My data may be used in encoded form for scientific reasons
- My data can be checked by the ethics committee
- to inform my doctor to participate in the study
- to be contacted by telephone for any data necessary for the study

Therefore, I agree to participate in the study

Patient signature and date

_________________________________________

DECLARATION OF CONSENT

I, the undersigned ______ _____declares:

- to have given the patient correctly and sufficiently all the necessary information
- to have given the patient a signed and dated copy of the informed consent signed by both the doctor and the patient

Date stamp and signature of the doctor

__________________ _____________________________
