# Supplementary material for: Prospective randomized study on the efficacy of three-dimensional reconstructions of bronchovascular structures on preoperative chest CT scan in patients who are candidates for pulmonary segmentectomy surgery: the PATCHES (Prospective rAndomized sTudy efficaCy of tHree-dimensional rEconstructions Segmentecomy) study protocol
Source: Trials. 2023 Sep 16;24:594. doi: 10.1186/s13063-023-07600-w (PMC10504737; doi:10.1186/s13063-023-07600-w)
Supplement: Supplementary file 2 — Additional file 2. Information sheet. [file 13063_2023_7600_MOESM2_ESM.docx]

| **Abteilung Gefäß- und Thoraxchirurgie**  **Vascular and Thoracic Surgery Division**  **Head physician**  **Dr. R Perkmann** |  |
| --- | --- |

PROSPECTIVE RANDOMIZED STUDY ON THE EFFICACY OF THREE-DIMENSIONAL RECONSTRUCTIONS OF BRONCHOVASCULAR STRUCTURES ON PREOPERATIVE CHEST CT IN PATIENTS WHO ARE CANDIDATES FOR PULMONARY SEGMENTECTOMY SURGERY

Dear patient you have been chosen to participate in a prospective multicenter randomized study as you will undergo thoracoscopic/robotic segmental lung resection surgery, i.e. the removal of a lung segment through small incisions and with the use of an optic connected to a camera that allows a complete view of the chest cavity.

We will now explain the meaning and objective of the study.

First of all, **prospective** means that the necessary data for this study will be recorded immediately by the medical staff, **multicenter** means that patients like you will be enrolled in other hospitals in different cities, and **randomized** means that neither the patient nor the surgeon but a random selection method determines to which group the patient belongs.

Before performing the surgery, you performed a chest CT scan with contrast medium and standard two-dimensional (2D) reconstructions were performed to study the lesion, the anatomy of the bronchi and the pulmonary venous and arterial vessels.

The surgeon's goal is to study the lung anatomy through preoperative CT and plan surgery. The three-dimensional (3D) reconstruction of anatomical structures, already widely used in endovascular surgery, could provide the surgeon with useful information, which reduces operating times, blood losses and conversions from minimally invasive procedure (thoracoscopy) to thoracotomic (traditional incision). The studies performed to date, however, are currently discordant, and an accurate analysis of the international literature concludes that there is insufficient scientific evidence to support the routine use of 3D reconstruction to reduce operating times, blood loss and conversions and that there is a need to perform further prospective randomized studies.

With this project we want to study the effectiveness of 3D reconstruction of preoperative CT to reduce operating times, blood loss and conversions after segmentectomy performed in thoracoscopy / robotics.

Therefore, after your signature of the informed consent, you will be enrolled in the study and a collaborator will record your personal data. The data will be encrypted and protected in a database. You will be randomly assigned to a study group.

The study involves enrolling patients in 2 different groups:

- group "A": reconstruction 2D+3D
- group "B": 2D reconstruction

At the end of the study, operating times, blood loss, the possible need for a conversion to traditional surgery, the duration of drainage and hospital stay are examined.

We expect at most a difference in conversions to traditional surgery of about 6%.

This study will be conducted in accordance with national legislation on self-funded trials and with the approval of the ethics committee.

The results will allow to define whether the 3D reconstruction of the preoperative chest CT is useful for the surgeon and for the patient.

Thank you for your attention and cooperation.
