# Supplementary material for: Prospective randomized study on the efficacy of three-dimensional reconstructions of bronchovascular structures on preoperative chest CT scan in patients who are candidates for pulmonary segmentectomy surgery: the PATCHES (Prospective rAndomized sTudy efficaCy of tHree-dimensional rEconstructions Segmentecomy) study protocol
Source: Trials. 2023 Sep 16;24:594. doi: 10.1186/s13063-023-07600-w (PMC10504737; doi:10.1186/s13063-023-07600-w)
Supplement: Supplementary file 3 — Additional file 3. Supplementary file. [file 13063_2023_7600_MOESM3_ESM.docx]

**Public Title**

Effectiveness of 3D reconstructions of anatomical structures on preoperative chest CT before sub-lobar resections for lung cancer.

**Scientific Title**

Prospective randomized study on the efficacy of three-dimensional reconstructions of bronchovascular structures on preoperative chest CT scan in patients who are candidates for pulmonary segmentectomy surgery: the PATCHES (Prospective rAndomized sTudy efficaCy of tHree-dimensional rEconstructions Segmentecomy) study protocol.

**Countries of Recruitment**

Italy, Germany, Belgium, Hong Kong, Palestine, Serbia, Spain, USA.

**Healthcare condition**

Lung cancer

**Interventions**

**Intervention Name**

Active Control group: 2D Reconstruction

**Intervention Description**

Before minimally invasive lung segmentectomy, preoperative chest CT scans are processed and evaluated with multi-plane (2D) reconstructions, according to the usual center protocol.

**Intervention Name**

Experimental group: 2D plus 3D Reconstruction

**Intervention Description**

Before minimally invasive lung segmentectomy, preoperative chest CT scans are processed and evaluated with multi-plane (2D) reconstructions plus a 3D reconstruction to study the lesion, the anatomy of the bronchi, and the pulmonary venous and arterial vessels.

**Inclusion Criteria**

• Segmentectomy performed through a minimally invasive approach (VATS or RATS).

• Pathologically proven NSCLC on the resected specimen.

• Age ≥18

• Signed written informed consent

**Exclusion criteria**

• Prior homolateral cardiothoracic surgery.

• Allergy or any other contraindication to iodinated contrast media.

• Segmentectomy performed through an open approach (thoracotomy).

• Histology different than NSCLC.

• Pregnancy

**Study Type**

Type of Study: Interventional

Study design: Randomized Controlled

Study Model: Parallel Assignment

Purpose: Treatment

**Date of First Enrollment**

August 1, 2023 (Anticipated)

**Sample Size**

Number of participants that the trial plans to enroll in total: 288.

**Recruitment Status**

Pending: participants are not yet being recruited or enrolled at any site

**Primary Outcomes**

1. Outcome Name: Margin- and disease-free resection

Method of measurement: millimeters of disease-free resection

Metric: final value

Method of aggregation: Median

Timepoint: 2 weeks following the end of the surgical procedure.

1. Outcome Name: Negative margin (R0) resection rate

Method of measurement: number of R0/R1 resections

Metric: final value

Method of aggregation: Proportion

Timepoint: 2 weeks following the end of the surgical procedure.

1. Outcome Name: Conversions from minimally invasive to thoracotomy procedure

Method of measurement: number of conversions

Metric: final value

Method of aggregation: Proportion

Timepoint: Intraoperative

**Key Secondary Outcomes**

1. Outcome Name: Operating times

Method of measurement: number of minutes

Metric: final value

Method of aggregation: Median

Timepoint: Intraoperative

1. Outcome Name: Intraoperative blood loss

Method of measurement: milliliters of blood loss

Metric: final value

Method of aggregation: Median

Timepoint: Intraoperative

1. Outcome Name: Intraoperative air leaks ~~and the use of sealants~~

Method of measurement: milliliters/min. of air leaks at the ventilator test ~~and number of~~

~~sealants~~

Metric: final value

Method of aggregation: Median

Timepoint: Intraoperative

1. Outcome Name: the use of sealants

Method of measurement: ~~milliliters of air leaks and~~ number of sealants

Metric: final value

Method of aggregation: Median

Timepoint: Intraoperative

1. Outcome Name: Incidence of 5-day Prolonged Air Leak (PAL)

Method of measurement: milliliters/min. of air leaks at digital chest drainage system

Metric: final value

Method of aggregation: proportion

Timepoint: every morning at 8 am until the chest drainage is removed.

1. Outcome Name: Postoperative hospitalization length

Method of measurement: number of days

Metric: time to event

Method of aggregation: median, stratified by thoracic surgery departments.

Timepoint: 3 weeks following the end of the surgical procedure.

**Ethics Review**

Status: Approved (N.: 1-2023)

Date of approval: 01.18.2023

Name of ethics committee: Ethics Committee Azienda Sanitaria Alto Adige (Bolzano, IT);

Contact details of ethics committee: R. Loss, MD

Address: L. Böhler street, 5

Telephone: +390471439039

E-mail: [rosanna.loss@sabes.it](mailto:rosanna.loss@sabes.it)

Each participating center submitted a request for approval by its ethics committee.

**Study Completion:** July 1, 2026 (Anticipated)

**IPD Sharing Statement**

Plan to Share IPD: No

**Protocol version number 1**

Issue date: 01.18.2023

**Funding**

Previously reported on page 1.

**Roles and Responsibilities**

Authors’ contributions

FZ, LB conceived of the study. FZ, LB, AK, and MDP initiated the study design and helped with implementation. LB and MDP provided statistical expertise in clinical trial design and are conducting the primary statistical analysis. All authors contributed to the refinement of the study protocol and approved the final manuscript.

Trial Sponsor: Central Hospital Bolzano

Sponsor’s Reference: EC Azienda Sanitaria Alto Adige

Contact name: R. Loss, MD

Address: L. Böhler street, 5

Telephone: +390471439039

E-mail: rosanna.loss@sabes.it

The funding source Central Hospital Bolzano had no role in the design of this study and will not have any role during its execution, analyses, interpretation of the data, or decision to submit results.

**Principal investigator and research physician**

FZ

**Roles and responsibilities:**

Design and conduct of PATCHES.

Preparation of protocol and revisions

Preparation of investigators brochure (IB) and CRFs [case report forms]

Organizing steering committee meetings

Managing CTO [clinical trials office]

Publication of study reports

**Steering committee** **(SC)**

FZ, LB, MDP, AK, FAA, MJ, MK, CSHN, PU, EP, MI

**Roles and responsibilities:**

Agreement of final protocol

All lead investigators will be steering committee members. One lead investigator per country will be nominated as the national coordinator.

Recruitment of patients and liaising with principal investigators.

Reviewing the progress of the study and, if necessary, agreeing to changes to the protocol and/or investigators’ brochure to facilitate the smooth running of the study.

**Trial management committee (TMC)**

LB, MDP, GC, AK

**Roles and responsibilities:**

Study planning

Organization of steering committee meetings

Provide the annual risk report ethics committee.

Responsible for trial master file

Budget administration and contractual issues with individual centers

Advice for lead investigators

Audit of monthly feedback forms and decide when site visits to occur.

Assistance with international review, board/independent ethics committee applications

Data verification

Randomization

**Data manager**

LB, MDP

**Roles and responsibilities:**

Maintenance of trial IT system and data entry

Data verification

**Lead investigators**

FAA, MJ, MK, CSHN, PU, EP, MI

**Roles and responsibilities:**

Identification, recruitment, data collection, and completion of CRFs, along with follow-up of study patients and adherence to study protocol and investigators brochure.
